# Bladder cancer stage and mortality: urban vs. rural residency

**DOI:** 10.1007/s10552-020-01366-1

**Published:** 2020-11-23

**Authors:** Marina Deuker, L. Franziska Stolzenbach, Claudia Collà Ruvolo, Luigi Nocera, Zhe Tian, Frederik C. Roos, Andreas Becker, Luis A. Kluth, Derya Tilki, Shahrokh F. Shariat, Fred Saad, Felix K.H. Chun, Pierre I. Karakiewicz

**Affiliations:** 1 Department of Urology, University Hospital Frankfurt, Frankfurt am Main, Germany; 2 Cancer Prognostics and Health Outcomes Unit, Division of Urology, University of Montréal Health Center, Montréal, Québec Canada; 3 Martini-Klinik Prostate Cancer Center, University Hospital Hamburg-Eppendorf, Hamburg, Germany; 4 Department of Urology, University of Naples Federico II, Naples, Italy; 5 Department of Urology and Division of Experimental Oncology, URI, Urological Research Institute, IBCAS San Raffaele Scientific Institute, Milan, Italy; 6 Department of Urology, Comprehensive Cancer Center, Medical University of Vienna, Vienna, Austria; 7 Institute for Urology and Reproductive Health, I.M. Sechenov First Moscow State Medical University, Moscow, Russia; 8 Department of Urology, University of Jordan, Amman, Jordan

**Keywords:** Bladder cancer, Social differences, Stage at presentation, Geographical disparities, Treatment rates

## Abstract

**Objective:**

Relative to urban populations, rural patients may have more limited access to care, which may undermine timely bladder cancer (BCa) diagnosis and even survival.

**Methods:**

We tested the effect of residency status (rural areas [RA < 2500 inhabitants] vs. urban clusters [UC ≥ 2500 inhabitants] vs. urbanized areas [UA, ≥50,000 inhabitants]) on BCa stage at presentation, as well as on cancer-specific mortality (CSM) and other cause mortality (OCM), according to the US Census Bureau definition. Multivariate competing risks regression (CRR) models were fitted after matching of RA or UC with UA in stage-stratified analyses.

**Results:**

Of 222,330 patients, 3496 (1.6%) resided in RA, 25,462 (11.5%) in UC and 193,372 (87%) in UA. Age, tumor stage, radical cystectomy rates or chemotherapy use were comparable between RA, UC and UA (all *p* > 0.05). At 10 years, RA was associated with highest OCM followed by UC and UA (30.9% vs. 27.7% vs. 25.6%, *p* < 0.01). Similarly, CSM was also marginally higher in RA or UC vs. UA (20.0% vs. 20.1% vs. 18.8%, *p* = 0.01). In stage-stratified, fully matched CRR analyses, increased OCM and CSM only applied to stage T1 BCa patients.

**Conclusion:**

We did not observe meaningful differences in access to treatment or stage distribution, according to residency status. However, RA and to a lesser extent UC residency status, were associated with higher OCM and marginally higher CSM in T1N0M0 patients. This observation should be further validated or refuted in additional epidemiological investigations.

## Introduction

Bladder cancer (BCa) stage and grade is strongly associated with cancer-specific mortality (CSM), therefore early diagnosis and treatment are imperative [1, 2]. Patients in rural areas may have more limited access to care and thus may be at risk for delayed bladder cancer diagnosis and treatment [3]. Many, predominantly historical, studies suggested that mortality rates were higher in rural areas compared to urban [4, 5]. To study that concept, we applied the official definition of rural and urban areas in the United States (US) according to the US Census Bureau and stratified our analyses according to urbanized areas (UA, ≥50,000 inhabitants) vs. urban clusters (UC, 2500–50,000 inhabitants) vs. rural areas (RA <2500 inhabitants) [6]. Within this stratification, we assessed potential differences in BCa stage at presentation, treatment patterns and cancer-specific mortality (CSM), as well as other cause mortality (OCM) in the most contemporary version of the SEER database. We hypothesized that RA residency status will be associated with higher stage at diagnosis and higher CSM, even after strict adjustment for OCM.

## Materials and methods

### Study population

The current SEER database samples 34.6% of the US population and approximates it in demographic composition and cancer incidence [7]. Within the SEER database (2004–2016), we identified patients ≥18 years old with histologically confirmed bladder cancer (International Classification of Disease for Oncology [ICD-O] site code C67.0–67.9). Cases identified only at autopsy or death certificate, were excluded. According to US Census Bureau definition, we included two types of metropolitan areas: urban areas (UA) and urban clusters (UC). UA were defined as areas with 50,000 or more inhabitants. UC were defined as areas with at least 2500 but fewer than 50,000 inhabitants. Rural areas (RA), conversely, were defined as all population, housing, and territory not included within UA or UC.

### Statistical analyses

Covariates in multivariate logistic regression analyses consisted of age at diagnosis, sex, race, grade, T-stage and N-stage and M-stage. Cumulative incidence plots assessed cancer-specific mortality (CSM), as well as other cause mortality (OCM) according to residency status (RA vs. UC vs. UA) in the overall cohort and in stage-specific analyses. In stage-specific, fully propensity score (PS)-adjusted analyses, two comparisons were made: (1) RA vs. UA, (2) UC vs. UA. For each individual comparison, PS-adjustment was applied for age, sex and socioeconomic status. Additional multivariate adjustment in competing risks regression (CRR) models was applied for age at diagnosis, sex, race, grade, surgical treatment type and chemotherapy [8]. Moreover, in CRR models, CSM estimates were adjusted for OCM and vice versa. In all statistical analyses, R software environment for statistical computing and graphics (R version 3.6.1) was used. All tests were two-sided with a level of significance set at *p* < 0.05.

## Results

### Descriptive characteristics of the study population

Within the SEER database, 222,330 bladder cancer patients of all stages were identified (Table 1). Of these, 3496 (1.6%) resided in RA, 25,462 (11.5%) resided in UC and 193,372 (87%) resided in UA. Mean age was comparable between RA, UC, and UA (72 vs. 72 vs. 73 years). High socioeconomic status was more prevalent in UC (35%) vs. RA (28.6%) vs. UA (23.4%). Married patients were most frequently recorded in RA (62.1%) followed by UC (60.5%) followed by UA (58.3%). Caucasian race was most frequently recorded in RA (96.6%) followed by UC (93.7%) followed by UA (88.3%).

**Table 1:** Patient and tumor characteristics of 222,330 bladder cancer patients of all stages, stratified according to rural or urban residency status, diagnosed within the SEER database from 2004 to 2016

	Cat/Stat	Overall 222,330	Rural Areas 3496 (1.6)	Urban Clusters 25,462 (11.5)	Urbanized Areas 193,372 (87)
Age at diagnosis	Mean (STE)	71.4 (0.025)	70.7 (0.192)	70.9 (0.073)	71.5 (0.027)
Age at diagnosis	Median (IQR)	72 (64–80)	72 (64–79)	72 (63–79)	73 (64–81)
Sex	Female	53,379 (24)	777 (22.2)	5649 (22.2)	46,953 (24.3)
	Male	168,951 (76)	2719 (77.8)	19,813 (77.8)	146,419 (75.7)
Race	White	197,907 (89)	3376 (96.6)	23,848 (93.7)	170,683 (88.3)
	Black	12,489 (5.6)	92 (2.6)	960 (3.8)	11,437 (5.9)
	Other	11,934 (5.4)	28 (0.8)	654 (2.6)	11,252 (5.8)
Socio economic status	1 quartile	55,288 (24.9)	1000 (28.6)	8993 (35.3)	45,295 (23.4)
	2–3-4 quartile	167,042 (75.1)	2496 (71.4)	16,469 (64.7)	148,077 (76.6)
Marital status	Married	130,378 (58.6)	2171 (62.1)	15,400 (60.5)	112,807 (58.3)
	Never Married	22,731 (10.2)	255 (7.3)	2166 (8.5)	20,310 (10.5)
	Separated/ Divorced	18,531 (8.3)	306 (8.8)	2286 (9)	15,939 (8.2)
	Unknown	18,103 (8.1)	276 (7.9)	1817 (7.1)	16,010 (8.3)
	Widowed	32,587 (14.7)	488 (14)	3793 (14.9)	28,306 (14.6)
Insurance status	Medicaid insured	12,848 (5.8)	298 (8.5)	1704 (6.7)	10,846 (5.6)
	Insured other	148,260 (66.7)	2243 (64.2)	16,780 (65.9)	129,237 (66.8)
	Uninsured	2673 (1.2)	55 (1.6)	417 (1.6)	2201 (1.1)
	Unknown	58,549 (26.3)	900 (25.7)	6561 (25.8)	51,088 (26.4)
T-stage	<=T1	173,890 (78.2)	2697 (77.1)	19,729 (77.5)	151,464 (78.3)
	T2	31,465 (14.2)	530 (15.2)	3797 (14.9)	27,138 (14)
	T3	8679 (3.9)	139 (4)	937 (3.7)	7603 (3.9)
	T4	8296 (3.7)	130 (3.7)	999 (3.9)	7167 (3.7)
N-stage	N0	205,748 (92.5)	3264 (93.4)	23,670 (93)	178,814 (92.5)
	N1	4338 (2)	55 (1.6)	463 (1.8)	3820 (2)
	N2	3807 (1.7)	58 (1.7)	434 (1.7)	3315 (1.7)
	N3	807 (0.4)	12 (0.3)	88 (0.3)	707 (0.4)
	Nx	7630 (3.4)	107 (3.1)	807 (3.2)	6716 (3.5)
M-stage	M0	209,506 (94.2)	3291 (94.1)	24,040 (94.4)	182,175 (94.2)
	M1	7887 (3.5)	130 (3.7)	897 (3.5)	6860 (3.5)
	MX	4937 (2.2)	75 (2.1)	525 (2.1)	4337 (2.2)
Grade	G1/G2	78,240 (35.2)	1383 (39.6)	9873 (38.8)	66,984 (34.6)
	G3/G4	105,459 (47.4)	1625 (46.5)	11,948 (46.9)	91,886 (47.5)
	Unknown	38,631 (17.4)	488 (14)	3641 (14.3)	34,502 (17.8)
Type of surgery	None	14,347 (6.5)	233 (6.7)	1786 (7)	12,328 (6.4)
	Partial cystectomy	2779 (1.2)	39 (1.1)	317 (1.2)	2423 (1.3)
	Radical cystectomy	20,353 (9.2)	343 (9.8)	2317 (9.1)	17,693 (9.1)
	TURB	183,607 (82.6)	2843 (81.3)	20,813 (81.7)	159,951 (82.7)
	Unknown	1244 (0.6)	38 (1.1)	229 (0.9)	977 (0.5)
Chemotherapy	No/Unknown	175,963 (79.1)	2725 (77.9)	19,451 (76.4)	153,787 (79.5)
	Yes	46,367 (20.9)	771 (22.1)	6011 (23.6)	39,585 (20.5)

### Stage at presentation and treatment rates

Tumor stage was comparable and absolute differences ranged from 0 to 1.2% (Table 1). Similarly, in RA vs. UC vs. UA, N0-stage was recorded in 93% vs. 93.0% vs. 92.5% and M0-stage was recorded in 94.1%, vs. 94.4% vs. 94.2%, respectively. Conversely, low tumor grade was more prevalent in RA (39.6%) vs. UC (38.8%) vs. UA (34.6%).

Rates of radical cystectomy were virtually the same: RA (9.8%), vs. UC (9.1%) vs. UA (9.1%). Conversely, chemotherapy use was marginally more frequently recorded in UC (23.6%) vs. RA (22.1%) vs. UA (20.5%).

### Logistic regression predicting advanced stage or treatment

In all six separate multivariate logistic regression analyses, predicting 1) advanced tumor stage (T_3–4_), 2) node positive stage (N_1–3_), 3) metastatic stage (M_1_), 4) high grade differentiation, 5) treatment with RC and 6) chemotherapy use, RA residency status did not predict the examined outcome (Table 2). Conversely, UC residency status was protective (OR 0.96, *p* = 0.003) from high grade differentiation and from node positive stage (OR 0.90, *p* = 0.03), when referenced to UA residency status. Moreover, UC residency status predicted higher rates of chemotherapy treatment (OR 1.21, *p* < 0.001), when referenced to UA residency status.

**Table 2:** Six separate logistic regression models predicting advanced tumor stage (T3–4, N1–3 or M1) high grade differentiation or treatment with radical cystectomy (RC) or chemotherapy according to residency status

	A. Predictors of advanced T-stage (T_3–4_)		B. Predictors of node positive stage (N_1–3_)		C. Predictors of metastatic stage M_1_	
	OR (95% CI)	p value	OR (95% CI)	p value	OR (95% CI)	p value
Urbanized Areas (Ref.)	1	–	1	–	1	–
Rural Areas	1.09 (0.94–1.25)	0.25	0.81 (0.62–1.04)	0.10	1.14 (0.93–1.38)	0.19
Urban Clusters	1.03 (0.98–1.09)	0.25	0.90 (0.82–0.99)	**0.03**	1.03 (0.94–1.11)	0.49

	D. Predictors of high grade differentiation		E. Predictors of treatment with RC		F. Predictors of treatment with chemotherapy	
	OR (95% CI)	p value	OR (95% CI)	p value	OR (95% CI)	p value
Urbanized Areas (Ref.)	1	–	1	–	1	–
Rural Areas	0.94 (0.87–1.01)	0.10	1.01 (0.87–1.17)	0.87	1.09 (0.99–1.18)	0.051
Urban Clusters	0.96 (0.93–0.99)	**0.009**	0.94 (0.89–1.00)	0.055	1.21 (1.17–1.25)	**<0.001**

p-values below 0.05 are displayed in bold

### Cumulative incidence plots of cancer-specific and other cause mortality

**Fig. 1:**
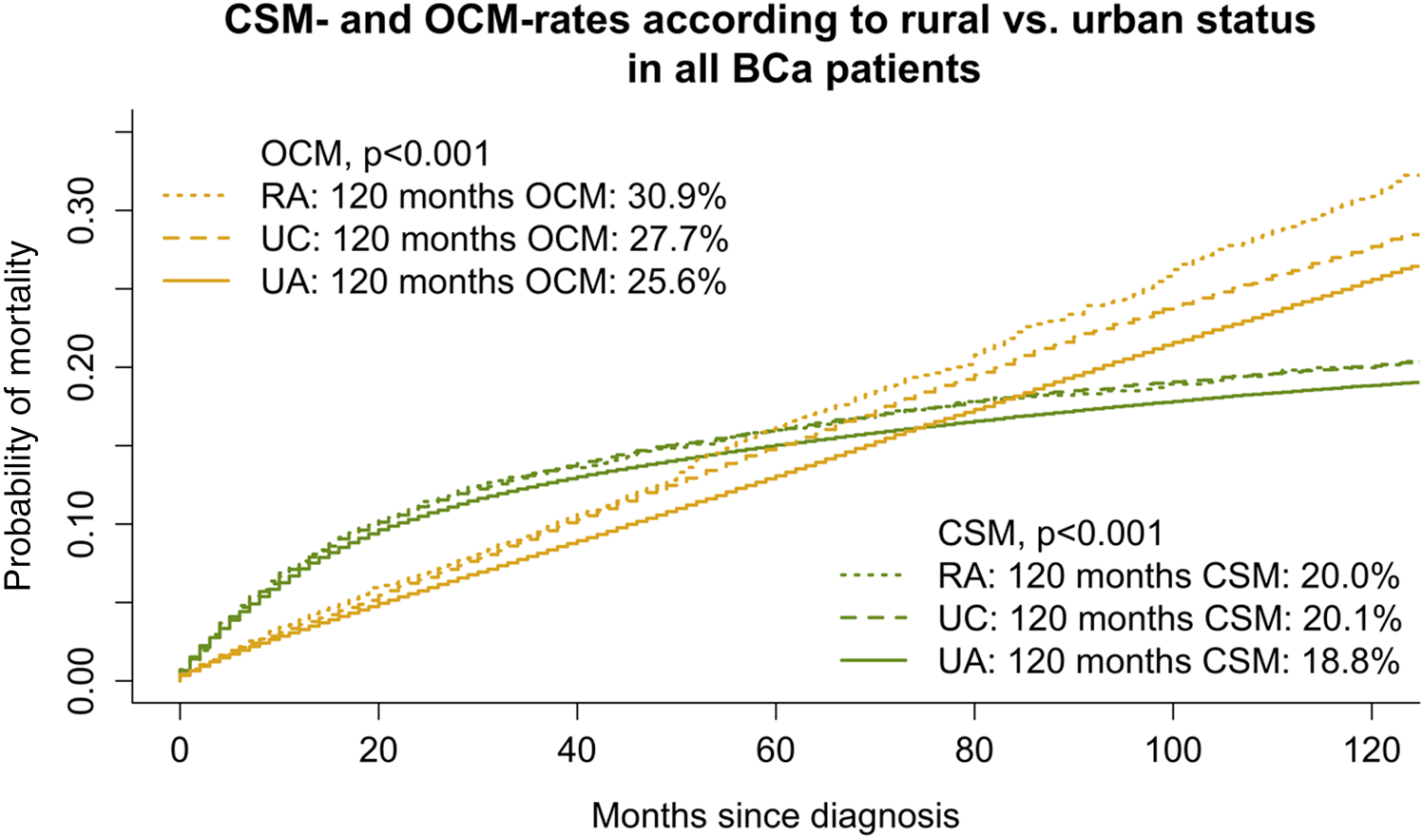
Cumulative incidence plots depicting cancer-specific mortality (CSM) and other cause mortality (OCM) according to residency status (rural area [RA] vs. urban cluster [UC] vs. urbanized area [UA]) in overall bladder cancer (BCa) patients

In the overall analyses, that included all tumor stages (Fig. 1), 10-year CSM rates according to RA vs. UC vs. UA status were 20.0% vs. 20.1% vs. 18.8% (*p* < 0.001). In stage-specific analyses, 10-year CSM rates were 12.2% vs. 11.6% vs. 10.7% (*p* = 0.001) in stage T1 BCa patients, according to respectively RA vs. UC vs. UA status. Conversely, in all other stage-specific analyses, no significant differences in CSM were recorded, according to RA vs. UC vs. UA residency status (data not shown).

In the overall analyses, 10-year OCM rates according to RA vs. UC vs. UA status were 30.9% vs. 27.7% vs. 25.6% (*p* < 0.01). Similarly, 10-year OCM rates in stage T1 BCa patients were 33.4% vs. 29.3% vs. 27.1% (p < 0.01), according to respectively RA vs. UC vs. UA residency status. Conversely, in all other stage-specific analyses, no significant differences in OCM were recorded, according to RA vs. UC vs. UA residency status (data not shown).

### Matched and multivariate competing risks regression analyses

(A) Cancer-specific mortality

**Fig. 2:**
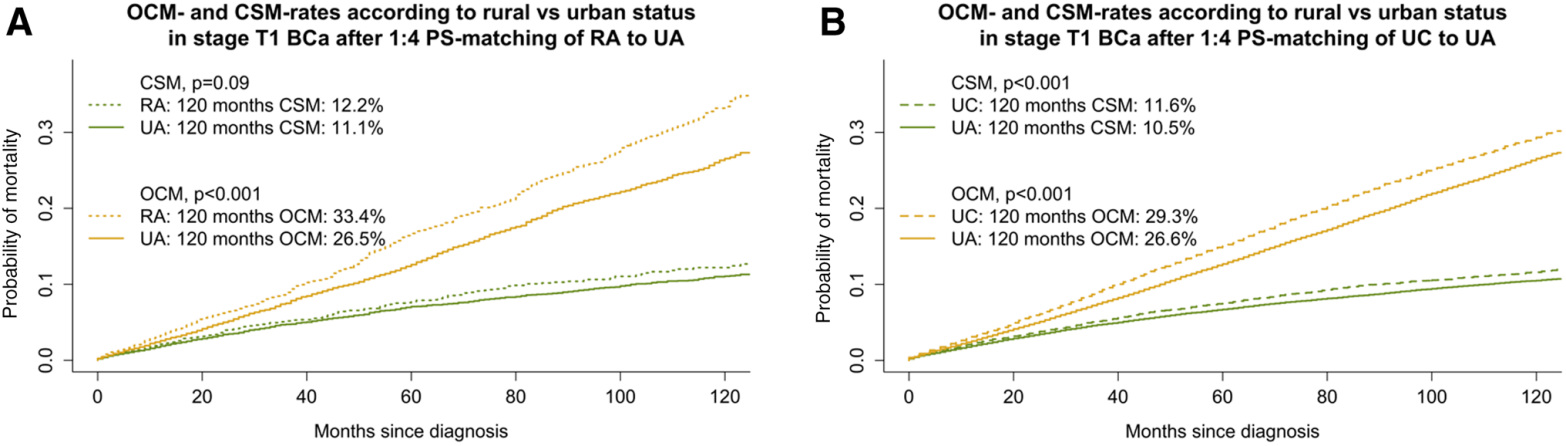
Cumulative incidence plots after 1:4 matching of (**a**) rural area (RA) residency status (n=2,651 RA) with urbanized area (UA) residency status (n=10,604 UC), or of (**b**) urban cluster (UC) residency status (n=19,437 UC) with UA (n=77,748), depicting cancer-specific mortality (CSM) and other cause mortality (OCM) in stage T1N0M0 BCa patients

After PS-matching and additional multivariate adjustments, CSM differences were recorded between 1) RA vs. UA and 2) UC vs. UA stage T1 BCa patients. Specifically, 10-year CSM rates were 12.2% vs. 11.1% (*p* = 0.09), according to RA vs. UA and 11.6% vs. 10.5% (*p* < 0.001), according to UC vs. UA residency status, respectively (Fig. 2). When referenced to UA in CRR (Table 3), RA and UC residency status were a risk factor for higher CSM in stage T1 BCa (HR 1.21, *p* = 0.02 and 1.17, *p* < 0.001). All other PS-matched and multivariate analyses, did not reveal statistically significant differences in CSM.

(B) Other cause mortality

**Table 3:** Two separate competing risks regression analyses, after matching of A) rural areas with urban areas and B) urbanized clusters with urban areas. Multivariate adjustment was made for Age at diagnosis, tumor grade, sex, race, socioeconomic status, surgical treatment and chemotherapy use

	CSM				OCM			
	HR univariate	p value	HR multivariate	p value	HR univariate	p value	HR multivariate	p value
A) Matched RA with UA								
UA	1.00 (Ref.)	–	1.00 (Ref.)	–	1.00 (Ref.)	–	1.00 (Ref.)	–
RA	1.14 (0.98–1.33)	0.09	1.21 (1.03–1.41)	0.019	1.28 (1.16–1.42)	<0.001	1.29 (1.17–1.43)	<0.001
B) Matched UA with UC								
UA	1.00 (Ref.)	–	1.00 (Ref.)	–	1.00 (Ref.)	–	1.00 (Ref.)	–
UC	1.13 (1.07–1.2)	<0.001	1.17 (1.1–1.24)	<0.001	1.17 (1.12–1.22)	<0.001	1.18 (1.14–1.23)	<0.001

After PS-matching and additional multivariate adjustments, OCM differences were also recorded between 1) RA vs. UA and 2) UC vs. UA stage T1 BCa patients. Specifically, 10-year OCM rates were 33.4% vs. 26.5% (*p* < 0.001) according to RA vs. UA and 29.3% vs. 26.6% (p < 0.001), according to UC vs. UA residency status, respectively (Fig. 2). When referenced to UA in CRR (Table 3), RA and UC residency status were a risk factor for higher OCM in stage T1 BCa (HR 1.29 and 1.18, both *p* < 0.001). All other PS-matched and multivariate analyses, did not reveal statistically significant differences in OCM.

## Discussion

We applied the official definition of rural and urban areas according to the US Census Bureau and stratified our analyses according to three different types of residential areas. Our work revealed several important observations.

*First* of all BCa patients in our analysis, only 1.6% accounted for RA residency status. Conversely, 11.5% were recorded in UC and 87% in UA residential areas, respectively. These rates differ substantially from the officially reported composition of the US according to the US Census Bureau [6]: In the year 2010, there were 486 UA and 3087 UC in the United States. UA accounted for 71.2% of the US population, while 9.5% resided in UC. Conversely, 19.3% resided in RA. Taking into account these major differences in the composition of the US population, the SEER database does not reflect the US in terms of urban vs. rural residency status. Rural regions of the US population are underrepresented in the SEER database. This fact is attributable to the composition of the SEER registries, that encompass mainly metropolitan regions and as such a majority of patients from UA or UC are registered in the database [9]. In consequence, it is difficult to analyze the effect of rural residency status within the SEER database, due to small numbers of RA observations. Ideally, future iterations of the SEER database should oversample rural areas, to better reflect the rural composition of the US.

*Second* despite the relatively small proportion of patients from rural areas, the size of the SEER database, allowed us to make important observations. RA residency status was not associated with meaningful differences in stage at presentation or with inferior access to care. This is conflicting with two previous reports from Monroe et al. (review from 1992) [10] and Hashibe et al. (*n* = 32,498 metropolitan and 4906 (13.1%) rural inhabitants, from 2014) [3]. Both investigators reported that rural cancer patients were diagnosed at a higher stage.

However, in our report, UC residency status was protective against N_1–3_ stage at presentation (HR: 0.90) and high grade differentiation (HR 0.96) compared to UA. Interestingly, chemotherapy rates were higher in UC than in UA (HR 1.21). To the best of our knowledge, no previous report examined differences in stage, treatment and mortality, according to substages of urban differentiation (UC vs. UA) and thus, we cannot compare our results to other reports.

*Third* in multivariately adjusted and matched analyses, we only observed marginal CSM differences (absolute difference of 1.2% in 10-year CSM rates: 20 vs. 18.8%) between RA and UA residency status. This is in agreement with our results regarding stage and grade distribution in rural patients, that were not worse for RA than UA patients. Similarly, patients residing within UC only exhibited marginal differences in CSM in comparison to UA residency status (absolute difference of 1.3% in 10-year CSM rates: 20.1% vs. 18.8%). Due to the large sample size of the SEER database, these marginal differences resulted in a statistically significant difference. Thus, prior works, that found higher CSM in RA [3–5] were numerically confirmed in our analyses. Nevertheless, it has to be emphasized that despite the statistical significance of our findings, the absolute differences in CSM are of unknown clinical importance. Moreover, in stage-specific analyses, this difference was only recorded in stage T1 patients. In higher tumor stages, no significant CSM differences between RA and UC or UA were registered. In consequence, rural area residency status predisposes to marginally higher CSM rates, but only in stage T1N0M0 patients. The overall result of no CSM difference is in concordance with three other studies [11–13]. Specifically, two of these studies focused on BCa and examined complications of radical cystectomy patients according to urban vs. rural status in a homogenous group of patients with the same insurance [11], as well as guideline adherence rates and mortality in non-muscle invasive BCa patients in a rural state [12]. However, no previous study examined CSM in stage-specific fashion. Therefore, we cannot compare our results of CSM in T1N0M0 patients with other reports. In consequence, more studies of CSM in RA BCa patients are warranted to further evaluate this potentially worrisome signal.

*In the final part of our analyses* we focused on OCM in RA vs. UC vs. UA patients of all stages. OCM rates demonstrated significant and meaningful differences according to residence in RA vs. UC vs. UA. Specifically, 10-year OCM rates were highest in RA (30.9%), followed by UC (27.7%) and UA (25.6%). The differences persisted and remained highly statistically significant, even after PS-matching for age, sex and socioeconomic status, as well as after multivariate adjustment for residual confounders and for the effect of competing cancer-specific mortality in stage T1N0M0 BCa patients: RA 33.4%, followed by UC 29.3% and UA 26.5%, but to a much lesser extent in all other BCa stages. In consequence, the observed OCM disadvantage in rural patients and to a lesser, albeit important and significant extent in UC patients, is worrisome. It indicates worse general health of rural area and urban cluster inhabitants with T1 BCa. It suggests higher prevalence of comorbidities, that are directly associated with OCM. Such OCM disadvantage in T1 RA and UC BCa patients warrants consideration, when treatments with important morbidity are considered. Radical cystectomy and chemotherapy for BCa represent such treatments. However, since our report represents the first signal suggesting an OCM disadvantage in RA and UC T1N0M0 BCa patients, further studies are clearly needed to validate or refute this observation.

*Taken together* our observations indicate that patients residing in rural areas, are not diagnosed at a later stage or with higher BCa grade. Moreover, they appear to benefit of available treatments, at least at the same rate as their urban counterparts. However, we recorded important and statistically significant OCM and marginal but statistically significant CSM differences, that were operational in stage T1N0M0 RA and UC patients, but not in other stage-specific analyses. OCM rates were highest in RA and were followed by UC and UA. The OCM differences potentially indicate worse general health of rural and urban cluster T1 BCa patients. Moreover, CSM differences suggest a marginal, albeit detectable disadvantage for RA and UC T1N0M0 BCa patients, relative to their UA counterparts. Further validation studies of CSM and OCM in RA and UC vs. UA BCa patients are clearly warranted. The accuracy of these observations may be limited by the relative paucity of rural patient composition, within the SEER database, and most likely within all other databases as well.

Despite multiple novel and important observations, several limitations may be applicable to our study. First and foremost, the number of patients with RA residency status was low and did not allow a proportional representation of the composition of the US population. Moreover, the retrospective, population-based nature of the SEER database did not allow us to control for some unavailable covariates and comorbidities. However, we adjusted all our analyses for OCM, which is a marker for the most important comorbidities, namely those resulting in death from other causes. Nevertheless, it would be of great interest to explore baseline comorbidity status in RA vs. UC vs. UA, that could potentially better explain the OCM disadvantage in RA vs. UC vs. UA. Limitations related to the retrospective, population-based nature of the SEER database, apply to this, as well as to other similar analyses that were based on the SEER database or on other similar large scale data repositories, such as National Cancer Data Base, National Inpatient Sample or National Surgical Quality Improvement Program.

## Conclusion

We did not observe meaningful differences in access to treatment or stage distribution, according to residency status. However, RA and to a lesser extent UC residency status, were associated with higher OCM and marginally higher CSM in T1N0M0 patients. This observation should be further validated or refuted in additional epidemiological investigations, that focus on RA and UC residents.

## Data Availability

SEER data are publicly available. Access to the SEER database is provided for researchers by the National Health Institute.

## Code availability

Custom code upon request.
